# Amyloid-*β* modulates APOE ε4 effects on cognition but not on targeted structural or functional connectivity measures over 2 years in pre-dementia adults

**DOI:** 10.3389/fnagi.2026.1889743

**Published:** 2026-07-31

**Authors:** Hongchun Wei, Tianhao Zhang, Zhigang Liang, Min Li, Min Kong, Maowen Ba

**Affiliations:** 1 Department of Neurology, The Affiliated Yantai Yuhuangding Hospital of Qingdao University, Yantai, Shandong, China; 2 Department of Neurology, Yantaishan Hospital, Yantai, Shandong, China; 3 Shandong Provincial Key Laboratory of Neuroimmune Interaction and Regulation, Yantai, China

**Keywords:** Alzheimer’s disease, amyloid-*β*, APOE ε4, cognitive decline, hippocampus-auditory network, structural MRI

## Abstract

**Background:**

The apolipoprotein E (APOE) ε4 allele is the strongest genetic risk factor for late-onset Alzheimer’s disease (AD), yet emerging evidence suggests its cognitive effects may be age- and pathology-dependent. Whether and how amyloid-*β* (A*β*) pathology modifies the longitudinal cognitive trajectory associated with APOE ε4 in the pre-dementia stage remains unclear.

**Methods:**

We conducted a 2-year longitudinal study in 70 pre-dementia adults from the Alzheimer’s disease Neuroimaging Initiative (ADNI), assessing APOE genotype, A*β* status via positron emission tomography (PET), multi-domain cognition, structural magnetic resonance imaging (MRI) volumes, and resting-state functional connectivity of a predefined hippocampus-auditory network. Linear mixed-effects models evaluated the three-way interaction of time, APOE ε4 carrier status, and A*β* status on longitudinal trajectories. Sensitivity and exploratory mediation analyses were performed.

**Results:**

A significant three-way interaction (Time × APOE ε4 × A*β* status) was observed for global cognitive decline (ADAS13: *β* = −4.54, *p* = 0.001, P_FDR = 0.003), indicating that A*β* pathology moderates the effect of APOE ε4 on cognitive trajectories. Post-hoc analyses revealed that among A*β*-positive individuals, APOE ε4 carriers exhibited a slower rate of cognitive decline compared to non-carriers, whereas no such difference was evident in A*β*-negative individuals. This moderating effect was robust to sensitivity analyses and was consistently observed across multiple cognitive domains (MMSE, CDRSB, FAQ, MoCA; all P_FDR < 0.01). However, no significant three-way interactions survived multiple comparison correction for regional brain volumes or hippocampus-auditory functional connectivity, though nominally significant trends were observed in middle temporal gyrus volume and specific temporal lobe connections. Longitudinal hippocampal atrophy did not mediate the observed association.

**Conclusion:**

A*β* pathology modifies the longitudinal cognitive trajectory associated with APOE ε4 in pre-dementia adults over a 2-year period. While consistent with the antagonistic pleiotropy framework, this observation requires independent replication, and alternative explanations including cognitive reserve and survivor bias warrant careful consideration.

## Introduction

Alzheimer’s disease (AD) is the leading cause of dementia worldwide. The ε4 allele of the apolipoprotein E gene (APOE ε4) is the strongest genetic risk factor for late-onset AD (Corder et al., 1993; Farrer et al., 1997), and is thought to accelerate pathogenesis by impairing A*β* clearance, exacerbating neuroinflammation, and promoting tau pathology (Verghese et al., 2011; Holtzman et al., 2000).

Recent evidence, however, reveals a more conditional role for APOE ε4. Large-scale studies have demonstrated age-dependent cognitive effects: in the UK Biobank (N > 320,000), ε4 homozygotes showed a cognitive advantage at age 40 that reversed after age 50 (Shibata et al., 2025), and ε4 heterozygotes in the Whitehall II cohort outperformed non-carriers at ages 45–55 before exhibiting accelerated decline after age 75 (Gharbi-Meliani et al., 2021). These patterns are consistent with the concept of antagonistic pleiotropy (Tuminello and Han, 2011), wherein a genetic variant confers advantages at certain life stages while increasing late-life vulnerability. In the context of AD pathology, APOE ε4 carriers with higher A*β* burdens have demonstrated superior visual working memory (Lu et al., 2021), and serum proteomic analyses suggest APOE ε4-dependent compensatory molecular pathways (Frick et al., 2024). Furthermore, APOE ε4 carriers may derive greater cognitive reserve benefits from modifiable lifestyle factors (O'Shea et al., 2024), and APOE ε4 amplifies the cognitive impact of non-APOE polygenic risk (Xu et al., 2024). Together, these findings position APOE ε4 as a conditional modifier whose net cognitive impact may be gated by age, pathological context, and environmental factors.

Despite this conceptual advance, critical questions remain in the prodromal stages of AD. It is unclear whether the interaction between APOE ε4 and A*β* pathology is observable in the earliest phases of cognitive change and whether it is detectable in brain structure or functional networks before widespread atrophy. According to the temporal model of AD pathophysiology, cognitive symptoms may precede detectable macrostructural atrophy (Jack et al., 2010; Bakhtiari et al., 2024), but the neural substrates of the APOE ε4-by-A*β* moderation effect remain poorly understood. The hippocampus and auditory network, both critically vulnerable in early AD, represent prime candidate circuits for such an investigation (Veldsman et al., 2021; Heise et al., 2014).

To address these gaps, we conducted a 2-year longitudinal study in pre-dementia individuals from ADNI. We employed linear mixed-effects models to test for a three-way interaction between time, APOE ε4 status, and A*β* status on trajectories of: (1) multi-domain cognition, (2) volumes of AD-vulnerable brain regions, and (3) resting-state functional connectivity within a targeted hippocampus-auditory network. Given the evidence for stage-dependent and A*β*-modulated cognitive effects of APOE ε4, we hypothesized that the longitudinal effect of APOE ε4 on cognition would be significantly moderated by A*β* status, and explored whether this moderated effect would be detectable in structural volumes or functional network connectivity.

## Materials and methods

### Study participants

Longitudinal data used in this study were obtained from the Alzheimer’s disease Neuroimaging Initiative (ADNI) database in September 2023. Participants were required to have baseline and longitudinal data on APOE genotype, amyloid-*β* (A*β*) positron emission tomography (PET), structural MRI, resting-state functional MRI (rs-fMRI), and comprehensive neuropsychological assessments.

To ensure maximum compatibility across measurements, we followed ADNI’s recommendations and included only the basic rs-fMRI version, excluding the advanced version of ADNI 3 due to incompatibility with ADNI-GO/2 protocols. Furthermore, we selected only participants with three consecutive years of rs-fMRI data (baseline, 12-month, and 24-month follow-ups).

Initially, 77 eligible participants were identified. After excluding six subjects with Alzheimer’s disease dementia and one subject whose diagnostic classification could not be determined, the final analytical sample comprised 70 pre-dementia participants (encompassing both cognitively normal older adults and those with mild cognitive impairment). The final sample was stratified into four groups based on APOE ε4 carriage and A*β* status: A*β*-negative ε4 non-carriers (*n* = 26), A*β*-negative ε4 carriers (*n* = 8), A*β*-positive ε4 non-carriers (*n* = 10), and A*β*-positive ε4 carriers (*n* = 26). All participants or their legal guardians provided written informed consent, and the study protocol was approved by the respective institutional review boards (see Supplementary Figure S1 for flow-chart showing cohorts’ selection, data preprocessing and analysis process).

### Clinical and biomarker assessments

At baseline and annual follow-ups, a standardized clinical evaluation was performed. Global cognition was assessed using the Mini-Mental State Examination (MMSE), Montreal Cognitive Assessment (MoCA), and the Alzheimer’s disease assessment scale–cognitive subscale 13 items (ADAS-Cog 13). Clinical disease stage was measured with the Clinical Dementia Rating Sum of Boxes (CDRSB). Daily functioning was evaluated with the Functional Activities Questionnaire (FAQ). Episodic memory was assessed using the Rey Auditory Verbal Learning Test immediate recall (RAVLT immediate) and the Logical Memory II delayed recall (LDELTOTAL).

APOE genotyping was performed, and participants were dichotomized into APOE ε4 carriers (≥1 ε4 allele) and non-carriers. The APOE ε4 genotype was coded as 0 (non-carrier) or 1 (carrier) for analyses.

Plasma p-tau181 levels were additionally assessed and included as a covariate in all models to account for potential confounding by tau pathology, given that tau-related neurodegeneration may independently influence cognitive decline.

### Neuroimaging acquisition and processing

*Structural MRI*: T1-weighted images were processed using standard pipelines for cortical reconstruction and subcortical segmentation. Intracranial volume (ICV) and volumes of regions of interest (ROIs)—including the hippocampus, whole brain, ventricles, entorhinal cortex, and middle temporal gyrus—were extracted for analysis.

*Resting-state fMRI*: resting-state functional MRI (rs-fMRI) data were acquired using blood-oxygen-level-dependent (BOLD) imaging during wakeful rest with eyes open. All rs-fMRI images sample for each participant consisted of 6,720 DICOM images. The sequence parameters were as follows: pulse sequence = GR, TR = 3,000 ms, TE = 30 ms, flip angle = 80°, data matrix = 64 × 64, pixel spacing X, Y = 3.31 mm and 3.31 mm, slice thickness = 3.33 mm, axial slices = 48, no slice gap, time points = 140. Rs-fMRI data preprocessing was performed using custom MATLAB scripts, SPM12, and RESTplus V1.24^1^. The preprocessing pipeline included the following steps: (1) converting DICOM format to NIfTI format; (2) removing the first 10 time points to allow for signal stabilization; (3) slice-timing correction; (4) head motion correction (realignment); (5) spatial normalization to standard Montreal Neurological Institute (MNI) space; (6) spatial smoothing with a 6-mm full-width at half maximum (FWHM) Gaussian kernel; (7) linear trend removal; (8) temporal band-pass filtering (0.01–0.08 Hz) to retain low-frequency fluctuations; and (9) nuisance regression to remove physiological artifacts, non-neuronal BOLD fluctuations (white matter and cerebrospinal fluid signals), and head motion parameters (Friston 24-parameter model) (Smith et al., 1999).

For seed-based functional connectivity (FC) analysis, we selected specific regions of interest (ROIs) from the CONN default atlas to minimize multiple comparison errors while focusing on core auditory processing areas (Fransson, 2005). Based on previous literature, we identified 6 ROIs encompassing the transverse temporal gyrus (Heschl’s gyrus, primary auditory cortex), anterior superior temporal gyrus, and posterior superior temporal gyrus (auditory association cortices) (Kaas and Hackett, 2000; Harasty et al., 1999; Wang et al., 2020). Additionally, we selected bilateral hippocampus as seed regions given their critical role in memory and early vulnerability in AD (Braak and Braak, 1991; Li et al., 2025) (see Supplementary Table S1 for ROI locations).

ROI-based FC was calculated to measure the functional relationship between the bilateral hippocampus and the selected auditory cortex regions. For each participant, mean time series were extracted from each ROI and correlated with time series from all other ROIs. The resulting correlation coefficients (r) were Fisher z-transformed to satisfy normality assumptions for statistical analysis. A global auditory network FC metric was computed as the mean of all 12 hippocampus-auditory connections. Additionally, individual FC values for each of the 12 predefined hippocampus-auditory connections were extracted for exploratory analyses.

*Amyloid PET*: [18F]Florbetapir PET scans were processed to create standardized uptake value ratio (SUVR) images normalized to the cerebellar gray matter reference region. Global cortical A*β* burden was quantified, and participants were classified as A*β*-positive (A+) or A*β*-negative (A−) using a threshold of SUVR ≥1.11 (Landau et al., 2013). In total, 70 participants were stratified into 34 A*β* − and 36 A*β* + groups. Detailed descriptions of PET image acquisition and processing are available at http://adni.loni.usc.edu/datasamples/pet/.

### Statistical analysis

All analyses were performed in R (v4.4.3). A two-sided *α* level of 0.05 was considered statistically significant unless otherwise specified for multiple comparisons.

*Baseline characteristics*: differences between APOE ε4 carriers and non-carriers were assessed using Student’s t-test or Mann–Whitney U test for continuous variables (presented as mean ± SD or median [IQR]) and chi-square or Fisher’s exact test for categorical variables [presented as *n* (%)]. Standardized mean differences (SMD) were calculated.

*Longitudinal cognitive and neuroimaging trajectories*: primary longitudinal analyses employed linear mixed-effects models (LMMs) fitted with the lmerTest package. The core model for each outcome (cognitive score, MRI volume, or FC value) was specified as:

*Outcome ~ Time × APOE ε4 × Aβ_status + Covariates + (1|RID).*

Here, time represents follow-up time in years (treated as continuous), APOE4 and A*β*_status are binary factors, and (1|RID) specifies a random intercept for each participant. The three-way interaction term (Time × APOE ε4 × A*β*_status) was the primary test of our hypothesis that A*β* pathology moderates the effect of APOE ε4 on longitudinal change. Covariates included baseline age, sex, years of education, global FDG-PET SUVR (when available), and plasma p-tau181 level. For structural MRI models, intracranial volume (ICV) was added as a covariate (except when ICV was the outcome). Statistical significance of fixed effects was assessed using Satterthwaite’s approximation for degrees of freedom. Post-hoc decomposition of significant interactions involved stratifying by A*β* status and fitting separate models within each stratum. Two-way interactions (Time × APOE ε4 and Time × A*β* status) were examined as secondary analyses.

*Mediation analysis*: an exploratory causal mediation analysis tested whether the effect of APOE ε4 on cognitive decline was mediated by hippocampal atrophy. We fitted three LMMs: (1) a total effect model (ADAS13 ~ Time×APOEε4 + covariates), (2) a mediator model (Hippocampus ~ Time×APOE ε4 + covariates), and (3) an outcome model (ADAS13 ~ Time×Hippocampus + Time×APOEε4 + covariates). The indirect effect (*a* × *b*) was computed, and its 95% confidence interval (CI) was estimated using non-parametric bootstrapping with 2,000 resamples. A CI excluding zero indicates significant mediation.

*Multiple comparison correction*: for analyses involving multiple related outcomes (7 cognitive tests, 6 structural ROIs, 12 FC connections), the false discovery rate (FDR) was controlled within each family of tests using the Benjamini-Hochberg procedure. Corrected *p* values are denoted as P_FDR.

*Sensitivity and robustness analyses*: the robustness of the primary cognitive finding (the three-way interaction) was assessed through three sensitivity analyses, each maintaining the same fixed-effects structure as the primary model: (1) We excluded observations identified as outliers (absolute standardized residual > 2.5) or high-leverage points, and refitted the full linear mixed-effects model (LMM); (2) We refitted the model using the nlme package with a first-order autoregressive [AR(1)] covariance structure to account for residual temporal autocorrelation, while keeping the fixed-effects formula identical; (3) We applied a robust linear mixed-effects estimator (rlmer from the robustlmm package) that is less sensitive to deviations from normality and the influence of outliers, using the same model formula. For the primary LMM, comprehensive model diagnostics were performed, including checks for convergence, residual normality (via Q–Q plots and the Shapiro–Wilk test), homoscedasticity, and multicollinearity (all variance inflation factors were <5).

## Results

### Participant characteristics

The analytical sample comprised 70 individuals in the pre-dementia stage of AD, encompassing both cognitively unimpaired adults and those with MCI. Baseline demographic, clinical, and neuroimaging characteristics, stratified by APOE ε4 carrier status, are detailed in Table 1. APOE ε4 carriers (*n* = 34) were significantly younger and had more years of formal education compared to non-carriers (*n* = 36) (both *p* < 0.05). As anticipated, carriers exhibited a substantially higher global amyloid-*β* burden, as measured by [18F]Flobetapir PET (median SUVR 1.28 vs. 1.04, *p* < 0.001). The two groups were well-balanced on all other key measures, including sex distribution, baseline scores across multiple cognitive domains, and regional brain volumes (all *p* > 0.05). The mean functional connectivity between bilateral hippocampi and a set of auditory cortex regions (including Heschl’s gyrus and anterior/posterior superior temporal gyrus) also did not differ between groups (*p* > 0.05).

**Table 1:** Baseline characteristics of participants stratified by APOE ε4 status.

Variable	APOEε4-noncarrier (*n* = 36)	APOEε4-carrier (*n* = 34)	*p*-value	SMD
Age, years, mean (SD)	73.71 (6.33)	70.17 (5.77)	**0.017**	0.584
Female, *n* (%)	18 (50.0)	18 (52.9)	0.816	0.059
Education, years, median [IQR]	16.00 [14.00, 16.00]	17.50 [16.00, 19.00]	**0.006**	0.671
A*β* positive, *n* (%)	10 (27.8)	26 (76.5)	**<0.001**	0.878
AV45 SUVR, median [IQR]	1.04 [1.01, 1.12]	1.28 [1.16, 1.43]	**<0.001**	0.958
FDG SUVR, mean (SD)	1.30 (0.11)	1.27 (0.10)	0.183	0.33
P-tau181 median [IQR]	22.49 [17.90, 27.78]	25.79 [20.46, 33.74]	0.09	0.51
MCI diagnosis, *n* (%)	23 (63.9)	25 (73.5)	0.446	0.209
MMSE, median [IQR]	29.00 [27.00, 29.00]	29.00 [28.00, 29.00]	0.772	0.04
ADAS13, median [IQR]	11.00 [8.00, 15.50]	11.00 [7.00, 15.00]	0.741	0.132
CDRSB, median [IQR]	1.00 [0.50, 2.00]	0.50 [0.00, 1.00]	0.1	0.306
FAQ, median [IQR]	0.00 [0.00, 3.50]	0.00 [0.00, 1.00]	0.124	0.276
MOCA, mean (SD)	24.00 (2.81)	24.67 (2.10)	0.272	0.269
LDELTOTAL, mean (SD)	8.67 (4.08)	10.12 (4.28)	0.154	0.347
RAVLT_immediate, mean (SD)	38.86 (10.95)	38.03 (9.20)	0.738	0.082
Hippocampus volume, mm^3^, mean (SD)	7262.27 (1072.52)	7558.03 (1021.50)	0.26	0.282
WholeBrain volume, mm^3^, mean (SD)	1059667.86 (104786.46)	1072460.61 (110070.31)	0.625	0.119
Ventricles volume, mm^3^, median [IQR]	32902.00 [21373.00, 44424.00]	27964.00 [18590.25, 41065.00]	0.34	0.224
Entorhinal volume, mm^3^, median [IQR]	3849.00 [3566.00, 4286.00]	3675.00 [3345.00, 4141.00]	0.315	0.173
Middle temporal gyrus volume, mm^3^, mean (SD)	20423.55 (2171.61)	20842.09 (3030.45)	0.523	0.159
ICV, mm^3^ mean (SD)	1555747.22 (166458.85)	1552103.82 (150775.89)	0.924	0.023
Auditory network FC, mean (SD)	0.22 (0.15)	0.18 (0.17)	0.361	0.219

Continuous variables are presented as mean ± standard deviation for normally distributed data or median (interquartile range) for skewed data (assessed by Shapiro–Wilk test). Group comparisons were performed using Student’s *t*-test for normal variables and Wilcoxon rank-sum test for non-normal variables.

SD, standard deviation; IQR, interquartile range; SMD, standardized mean difference; SUVR, standardized uptake value ratio; AV45, [18F]florbetapir; FDG, fluorodeoxyglucose; PET, positron emission tomography; p-tau181, plasma phosphorylated tau 181; MCI, mild cognitive impairment; MMSE, Mini-Mental State Examination; ADAS13, Alzheimer’s disease assessment scale–cognitive subscale 13 items; CDRSB, clinical dementia rating sum of boxes; FAQ, functional activities questionnaire; MoCA, montreal cognitive assessment; LDELTOTAL, logical memory II delayed recall; RAVLT, rey auditory verbal learning test; ICV, intracranial volume; FC, functional connectivity.

The four-group cross-tabulation of APOE ε4 carrier status and Aβ status yielded the following: A*β*-negative ε4 non-carriers (*n* = 26), A*β*-negative ε4 carriers (*n* = 8), Aβ-positive ε4 non-carriers (*n* = 10), and A*β*-positive ε4 carriers (*n* = 26).

Missing data: FDG-PET SUVR (3/70, 4.3%); p-tau181 (8/70, 11.4%); MMSE (1/70, 1.4%); ADAS13 (1/70, 1.4%); CDRSB (1/70, 1.4%); FAQ (1/70, 1.4%); MoCA (1/70, 1.4%); Logical Memory II delayed recall (1/70, 1.4%); RAVLT immediate (2/70, 2.9%); Hippocampus volume (5/70, 7.1%); Whole brain volume (2/70, 2.9%); Ventricles volume (3/70, 4.3%); Entorhinal cortex volume (5/70, 7.1%); Middle temporal gyrus volume (5/70, 7.1%).Bold values indicate statistically significant differences between APOE ε4 carriers and non-carriers (*p* < 0.05).

### A*β* status moderates APOE ε4-associated cognitive trajectories

Longitudinal analysis of cognitive decline over 2 years revealed significant three-way interactions between time, APOE ε4 status, and A*β* status across multiple cognitive domains (Table 2). For the primary outcome ADAS13, the three-way interaction was highly significant (*β* = −4.54, SE = 1.37, *t* = −3.33, *p* = 0.001), indicating that the effect of APOE ε4 on cognitive decline rates differed significantly between A*β*-positive and A*β*-negative individuals. This interaction remained significant after FDR correction across seven cognitive domains (P_FDR = 0.003).

**Table 2:** Three-way interaction (time × APOE ε4 × A*β* status) on cognitive decline over 2 years.

Cognitive measure	*β*	SE	*t*	*p*	P_FDR	Interpretation
MOCA	2.969	0.66	4.5	<0.001	<0.001	**
CDRSB	−1.103	0.318	−3.47	0.001	0.003	**
ADAS13	−4.542	1.366	−3.33	0.001	0.003	**
MMSE	1.969	0.658	2.99	0.003	0.006	**
FAQ	−2.449	0.875	−2.8	0.006	0.008	**
RAVLT_immediate	4.161	2.091	1.99	0.049	0.057	†
LDELTOTAL	1.764	1.151	1.53	0.128	0.128	—

Linear mixed-effects models were fitted for each cognitive outcome, incorporating the three-way interaction term (Time × APOE ε4 × A*β* status) along with covariates for age, sex, education, FDG uptake, and plasma p-tau levels. The table reports the coefficient estimates (*β*), standard errors (SE), *t*-statistics, nominal *p*-values, and FDR-corrected *p*-values (P_FDR) for the three-way interaction term.

**P_FDR < 0.01; ^†^*p* < 0.05 but P_FDR > 0.05.

*β*, regression coefficient; SE, standard error; FDR, false discovery rate; ADAS13, Alzheimer’s Disease assessment scale-cognitive subscale 13 items; CDRSB, clinical dementia rating sum of boxes; FAQ, functional activities questionnaire; LDELTOTAL, logical memory II delayed recall; MMSE, mini-mental state examination; MoCA, montreal cognitive assessment; RAVLT, rey auditory verbal learning test.

Significant three-way interactions surviving FDR correction were consistently observed for global cognition and screening measures: MMSE (*β* = 1.97, *p* = 0.003, P_FDR = 0.006), CDRSB (*β* = −1.10, p = 0.001, P_FDR = 0.003), FAQ (*β* = −2.45, *p* = 0.006, P_FDR = 0.008), and MoCA (*β* = 2.97, *p* < 0.001, P_FDR < 0.001). The RAVLT immediate recall showed a nominal trend (*β* = 4.16, *p* = 0.049, P_FDR = 0.057), while logical memory (LDELTOTAL) did not show significant moderation (*p* = 0.128).

**Figure 1:**
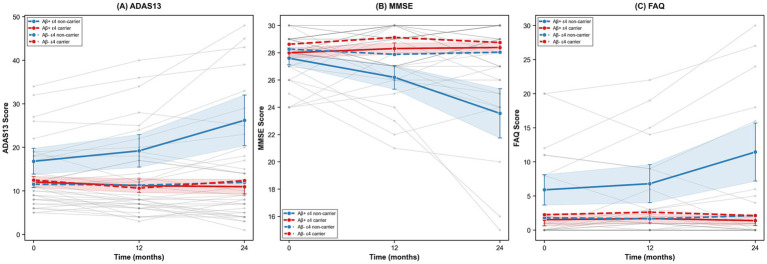
Modulation of APOE ε4 effects on cognition by amyloid-*β* (A*β*) pathology. Longitudinal trajectories from linear mixed-effects models with a three-way interaction (Time × APOE ε4 × A*β* status) are shown for **(A)** ADAS13, **(B)** MMSE, and **(C)** FAQ. Within each panel, solid lines represent A*β*-positive participants and dashed lines represent A*β*-negative participants, with blue indicating APOE ε4 non-carriers and red indicating APOE ε4 carriers. Shaded areas denote ±1 standard error (SE) for the A*β*-positive groups. Individual participant trajectories (grey lines with dots, *α* = 0.3) are overlaid for the A*β*-positive groups to visualize raw data distribution. All models were adjusted for age, sex, education, FDG-PET SUVR, and plasma p-tau181 levels.

**Figure 2:**
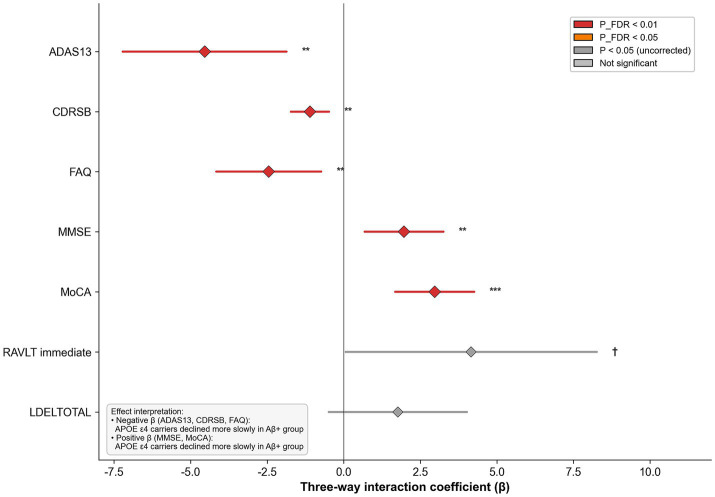
Forest plot showing three-way interaction coefficients (Time × APOE ε4 × A*β* status) for seven cognitive outcomes. Error bars represent 95% confidence intervals. **P_FDR < 0.01; *P_FDR < 0.05; ^†^*p* < 0.05 (uncorrected). Negative *β* values for ADAS13, CDRSB, and FAQ indicate slower decline in APOE ε4 carriers within the A*β* + group. Positive *β* values for MMSE and MoCA indicate better preserved cognition in APOE ε4 carriers within the A*β* + group.

Post-hoc stratification by amyloid status clarified this moderation effect. As visualized in Figure 1, the association between APOE ε4 carriage and attenuated cognitive decline was predominantly observed in the A + subgroup. Within the A*β*-positive group, APOE ε4 carriers exhibited significantly slower rates of decline on ADAS13, MMSE, and FAQ compared to non-carriers. In contrast, cognitive trajectories between APOE ε4 carriers and non-carriers were not significantly different within the A − stratum, indicating that the protective-appearing effect of APOE ε4 is contingent upon the presence of A*β* pathology (Figure 2).

While the primary focus of this study was on three-way interactions, we also conducted secondary analyses examining two-way interactions between Time × APOE ε4 and Time × A*β* status. These results are presented in Supplementary Table S2 and visualized in Supplementary Figure S2.

### Absence of robust modulating effects on brain structure and targeted functional connectivity

We next examined whether the observed cognitive interaction was paralleled by changes in neuroimaging markers. For volumetric measures from structural MRI, no regional three-way interactions (Time × APOE ε4 × A*β* status) survived FDR correction (all P_FDR > 0.05; Supplementary Table S3). However, nominally significant interactions (uncorrected *p* < 0.05) were observed for middle temporal gyrus volume (*β* = 725.79, *p* = 0.007, P_FDR = 0.070) and ventricular expansion (*β* = −2600.65, *p* = 0.012, P_FDR = 0.071), suggesting potential trends that did not withstand multiple comparison correction. Similarly, analysis of 12 predefined hippocampus-auditory cortex functional connections showed no significant interactions after FDR correction (all P_FDR > 0.15; Supplementary Table S4). A nominally significant association was observed for the right hippocampus–left posterior superior temporal gyrus connection (*β* = −0.238, *p* = 0.013, P_FDR = 0.151), with two additional connections showing marginal trends (right hippocampus–right Heschl’s gyrus: *p* = 0.064; left hippocampus–right posterior STG: *p* = 0.088). Notably, the nominally significant structural MRI interactions were directionally consistent with the cognitive findings (i.e., relative preservation among A*β*-positive ε4 carriers), while the nominally significant functional connectivity interaction for the right hippocampus–left posterior STG connection was in the opposite direction, suggesting greater connectivity decline among A*β*-positive ε4 carriers.

### Sensitivity analyses confirm robustness of the primary cognitive finding

The significance and direction of the key three-way interaction on ADAS13 scores remained consistent across all sensitivity analyses designed to address potential model violations (Table 3). The effect was virtually unchanged when accounting for residual autocorrelation (*β* = −4.49, *p* = 0.004) and was confirmed using a robust estimator less sensitive to outliers and non-normality (*β* = −4.18, *p* < 0.01). Excluding observations identified as high-leverage points also yielded a consistent estimate (*β* = −4.31, *p* = 0.005).

**Table 3:** Sensitivity analyses for the primary three-way interaction (time × APOE ε4 × A*β* status) on ADAS13 scores.

Model specification	*β*	SE	*t*/*z* value	*p*	*N* (observations)
Primary model (standard LMM)	−4.50	1.45	−3.11	0.002	164
1. Excluding outliers/influential points	−4.31	1.49	−2.89	0.005	164
2. With AR(1) covariance (autocorrelation)	−4.49	1.52	−2.95	0.004	164
3. Robust linear mixed-effects model (RLMER)	−4.18	1.26	−3.31	<0.01	164

*β*, regression coefficient; SE, standard error; t/z, t-statistic or z-statistic; P, statistical significance; N, number of observations; LMM, linear mixed-effects model; AR(1), first-order autoregressive covariance structure; rlmer, robust linear mixed-effects regression. All models evaluated the three-way interaction (Time* APOE ε4*Aβ status) on ADAS13 scores. The primary model used standard LMM with random intercepts. Sensitivity analyses included: (1) exclusion of outliers and influential points; (2) AR(1) covariance structure to account for temporal autocorrelation; (3) robust estimation to minimize influence of outliers and non-normality.

To further assess the robustness of our primary findings, we repeated the three-way interaction analysis using continuous AV45 SUVR (centered) instead of dichotomized A*β* status. The continuous SUVR analysis yielded results that were entirely consistent with the dichotomous approach: all seven cognitive measures showed directionally concordant three-way interactions (Time × APOE ε4 × AV45 SUVR), with four measures (ADAS13, MMSE, CDRSB, and MOCA) remaining statistically significant after FDR correction (all P_FDR < 0.003; Supplementary Table S5). This convergence across dichotomized and continuous A*β* operationalizations substantially strengthens the primary finding.

### Exploratory analysis: hippocampal atrophy did not mediate the APOE ε4 effect

An exploratory causal mediation analysis was performed to test whether longitudinal hippocampal atrophy mediated the effect of APOE ε4 genotype on cognitive decline. The results indicated no significant mediation (Supplementary Table S6). While the total effect of APOE ε4 on the rate of ADAS13 increase was significant, the specific indirect effect through hippocampal volume change was negligible, with its bootstrapped 95% confidence interval encompassing zero. The proportion of the total effect mediated by hippocampal atrophy was minimal (0.6%). These findings suggest that, within the 2-year observation window of this cohort, the association between APOE ε4 and cognitive decline was not significantly explained by concurrent macrostructural changes in hippocampal volume.

## Discussion

In this 2-year longitudinal study of pre-dementia individuals, we investigated whether cerebral A*β* pathology modifies the longitudinal effects of the APOE ε4 allele on cognitive and neuroimaging trajectories. Our key finding is that the effect of APOE ε4 on cognitive decline is critically moderated by A*β* pathology. Specifically, APOE ε4 carriers with established A*β* pathology exhibited a slower rate of cognitive decline compared to non-carriers. This result challenges the traditional view of APOE ε4 as a uniformly detrimental risk factor across all disease stages and instead supports an emerging model wherein its phenotypic impact is conditional, manifesting primarily in the context of co-existent A*β* pathology.

### APOE ε4, a*β* pathology, and the conditional nature of cognitive decline

Our finding—that A*β* pathology moderates the cognitive trajectory of APOE ε4 carriers in the pre-dementia stage—appears paradoxical but is consistent with the antagonistic pleiotropy framework (Tuminello and Han, 2011) and with recent evidence that APOE ε4 modulates amyloid-cognition relationships in preclinical stages (Brugulat-Serrat et al., 2023; Lim et al., 2015). This framework posits that the cognitive effects of APOE ε4 vary systematically across the lifespan and disease continuum. In healthy adults, ε4 is associated with cognitive advantages, including enhanced episodic memory (Mondadori et al., 2007), superior verbal fluency (Alexander et al., 2007), and broader attentional deployment (Rusted et al., 2013), suggesting underlying biological programs that support efficient neural processing. Intriguingly, a similar “reversal” of effect direction is observed at the advanced disease stage, where ε4 homozygotes with established AD exhibit slower cognitive decline than non-carriers (Frisoni et al., 1995; Hoyt et al., 2005; Almkvist et al., 2022). Our study bridges these two extremes by demonstrating that the moderating effect of A*β* on ε4-associated trajectories is already operative during the critical pre-dementia transition.

The stage-dependent impact of APOE ε4 may be explained by its cell type-specific actions. A hierarchical cascade model proposes that neuronal APOE ε4 acts as an upstream initiator of pathology (Blumenfeld et al., 2024): its stress-induced upregulation leads to neurotoxic C-terminal fragments, mitochondrial dysfunction, and synaptic loss, while also enhancing inter-neuronal tau spread via heparan sulfate proteoglycans (Nelson et al., 2023). In contrast, astrocytic APOE ε4 primarily drives cerebrovascular dysfunction and neuroinflammation, contributing to blood–brain barrier leakage (Jackson et al., 2022; Xiong et al., 2023). The net cognitive effect of ε4 may thus reflect a shifting balance between these detrimental pathways and potential compensatory responses in other cellular compartments across different disease stages.

Beyond direct cellular mechanisms, alternative explanations warrant consideration. The observed moderation effect may be influenced by cognitive reserve: ε4 carriers who reach the A*β* + stage may possess greater neural compensation capacity, as suggested by evidence of increased frontal activation during cognitive tasks (Lu et al., 2021). Survivor bias is also a possibility, wherein A*β* + ε4 carriers who remain in longitudinal studies constitute a uniquely resilient subgroup. Importantly, these interpretations are not mutually exclusive and may operate synergistically with the cell type-specific mechanisms described above.

The conditional nature of APOE ε4’s cognitive effects underscore the critical importance of biomarker context (particularly A*β* status) when evaluating genetic risk in both clinical practice and trial design. Furthermore, the cell type specificity of APOE ε4 action suggests that future therapeutic strategies may need tailoring: interventions targeting neuronal APOE ε4 could be pivotal for preventing neurodegeneration, while those targeting astrocytic APOE ε4 might be more effective for preserving cerebrovascular integrity.

### Neuroimaging findings and hippocampal mediation analysis

A second key finding was that the observed effect of APOE ε4 on cognitive slopes was not significantly mediated by concurrent rates of hippocampal atrophy over the 2-year observation window. The indirect effect was negligible, and the proportion of the total effect explained by hippocampal volume change was minimal (0.6%). This suggests that in this early disease stage, the APOE ε4-by-A*β* interaction influences cognition through mechanisms that are either more subtle than gross structural loss or operate on a different, potentially earlier, temporal scale. While APOE ε4 is consistently associated with accelerated hippocampal atrophy in AD (Mori et al., 2002), and some studies report a stronger coupling between hippocampal atrophy and memory decline in ε4 carriers (Gorbach et al., 2020), the timing of these relationships may be stage-dependent. A recent study highlighted that the effect of APOE ε4 on hippocampal atrophy is strongest in the prodromal (MCI) phase but is particularly driven by amyloid-positive individuals (Wang et al., 2023). Our null mediation result does not contradict the established link between APOE ε4 and atrophy but may indicate that over a relatively short 2-year period in a heterogeneous pre-dementia group, the variance in cognitive decline attributable to APOE ε4 is not yet fully captured by, or transmitted through, measurable macrostructural hippocampal change. Instead, the cognitive effects may be more directly related to synaptic dysfunction, altered network connectivity, or other A*β*- and tau-related toxicities that precede volumetric loss (Selkoe, 2002; DeKosky and Scheff, 1990).

The absence of a significant interaction effect on hippocampus-auditory network functional connectivity, despite its *a priori* selection as an early vulnerable circuit, further supports the specificity of our primary finding. It suggests that the moderated cognitive effect is not broadly reflected in the functional integrity of this specific network at this disease stage. This aligns with the temporal model of AD biomarkers, where cognitive symptoms often emerge from a complex pathophysiology that may not yet be fully mirrored in every imaging modality (Mila-Aloma et al., 2025).

Notably, while no structural or functional imaging markers survived multiple comparison correction, several nominally significant trends warrant consideration. The middle temporal gyrus, a region critically involved in semantic memory and language processing, showed a trend-level three-way interaction (*p* = 0.007, P_FDR = 0.070). This finding is biologically plausible given the temporal lobe’s early vulnerability in AD and its dense connectivity with the hippocampus (de Flores et al., 2022). Similarly, ventricular expansion—a sensitive but non-specific marker of brain atrophy—showed a comparable trend (*p* = 0.012, P_FDR = 0.071). In the functional domain, the right hippocampus–left posterior superior temporal gyrus connection exhibited a nominal interaction (*p* = 0.013), consistent with the structural observation in temporal regions. The posterior superior temporal gyrus participates in auditory language comprehension and has been implicated in early AD-related network disruptions (Pistono et al., 2021). These convergent trends across structural and functional modalities in temporal lobe-related measures suggest that the APOE ε4–A*β* interaction may begin to manifest in temporal lobe integrity before reaching statistical thresholds for multiple comparisons.

Interestingly, the nominally significant structural MRI trends (middle temporal gyrus volume and ventricular expansion) were directionally consistent with the cognitive findings, suggesting relative preservation among A*β*-positive ε4 carriers. In contrast, the nominally significant functional connectivity interaction for the right hippocampus–left posterior STG connection was in the opposite direction, indicating greater connectivity decline among A*β*-positive ε4 carriers. This dissociation between structural preservation and functional connectivity decline may reflect distinct temporal dynamics of APOE ε4-mediated pathophysiology, wherein macrostructural changes may be partially buffered by compensatory mechanisms even as network-level connectivity begins to deteriorate.

However, we emphasize that these observations are hypothesis-generating and require independent validation in larger cohorts with extended follow-up periods.

### Strengths and limitations

The strengths of this study include its well-characterized pre-dementia cohort, the use of a comprehensive multi-modal biomarker dataset (A*β*-PET, MRI, rs-fMRI), and the application of rigorous longitudinal mixed-effects models that account for within-subject correlations. However, several limitations must be acknowledged. The 2-year follow-up period, while sufficient to detect cognitive slopes, may be too short to observe the full unfolding of structural changes or to definitively exclude survivor effects, particularly given that rapid cognitive decline in AD is influenced by multiple interacting risk factors (Song et al., 2018). Our sample size, while adequate for detecting the primary three-way interaction, may have limited power for detecting smaller neuroimaging effects or mediation relationships. We did not incorporate tau-PET, which would have allowed more precise characterization of how tau pathology interacts with APOE ε4 and A*β* status. Finally, the cross-tabulation of APOE ε4 and A*β* status resulted in uneven group sizes, with the A*β*-positive ε4 non-carrier group being notably small (*n* = 10). Although sensitivity analyses confirmed the robustness of our cognitive findings, this imbalance limits statistical power for subgroup comparisons and raises the possibility that the observed effects in this subgroup could be influenced by a small number of individuals. Our findings require independent replication in larger, balanced cohorts.

A post-hoc power analysis indicated that the minimum detectable effect size for the three-way interaction term at *α* = 0.05 and 80% power was Cohen’s *f*^2^ ≈ 0.076 (Cohen’s *d* ≈ 0.55). The observed effect size for the ADAS13 three-way interaction (Cohen’s *d* ≈ 1.45) substantially exceeded this threshold, with an estimated post-hoc power of approximately 91%. This confirms that the primary cognitive finding was adequately powered despite the modest overall sample size. However, we acknowledge that the power to detect smaller neuroimaging effects or the mediation pathway was limited, as evidenced by the non-significant structural and functional connectivity interactions and the null mediation result.

## Conclusion

In conclusion, our study demonstrates that in pre-dementia individuals, the effect of APOE ε4 on the rate of cognitive decline is not a fixed trait but is powerfully moderated by A*β* pathology. The finding that APOE ε4 carriers in the A*β* + group exhibited slower cognitive decline challenges the traditional view of APOE ε4 as a uniform risk factor and underscores the importance of considering biomarker context when evaluating genetic risk in clinical practice and trial design. Furthermore, the lack of mediation through hippocampal atrophy over 2 years suggests that the early cognitive impact of this gene-pathology interaction may be driven by mechanisms other than, or preceding, overt structural degeneration. These results contribute to a more nuanced understanding of APOE ε4’s role in AD progression, highlighting it as a conditional genetic factor whose clinical manifestation depends on the co-pathology landscape, particularly during the critical transitional stage of pre-dementia.

## Data Availability

Publicly available datasets were analyzed in this study. This data can be found here: the data used in this study were obtained from the Alzheimer’s disease neuroimaging initiative (ADNI) database (https://adni.loni.usc.edu). Data were downloaded in September 2023 following the standard ADNI data access procedures. No specific accession numbers apply to this publicly available dataset.

## Footnotes

1
http://www.restfmri.net
