# Supplementary material for: Amyloid-β modulates APOE ε4 effects on cognition but not on targeted structural or functional connectivity measures over 2 years in pre-dementia adults
Source: Front Aging Neurosci. 2026 Jul 31;18:1889743. doi: 10.3389/fnagi.2026.1889743 (PMC13472819; doi:10.3389/fnagi.2026.1889743)

FigureS1: Flow-chart showing cohorts selection, data preprocessing and analysis process.

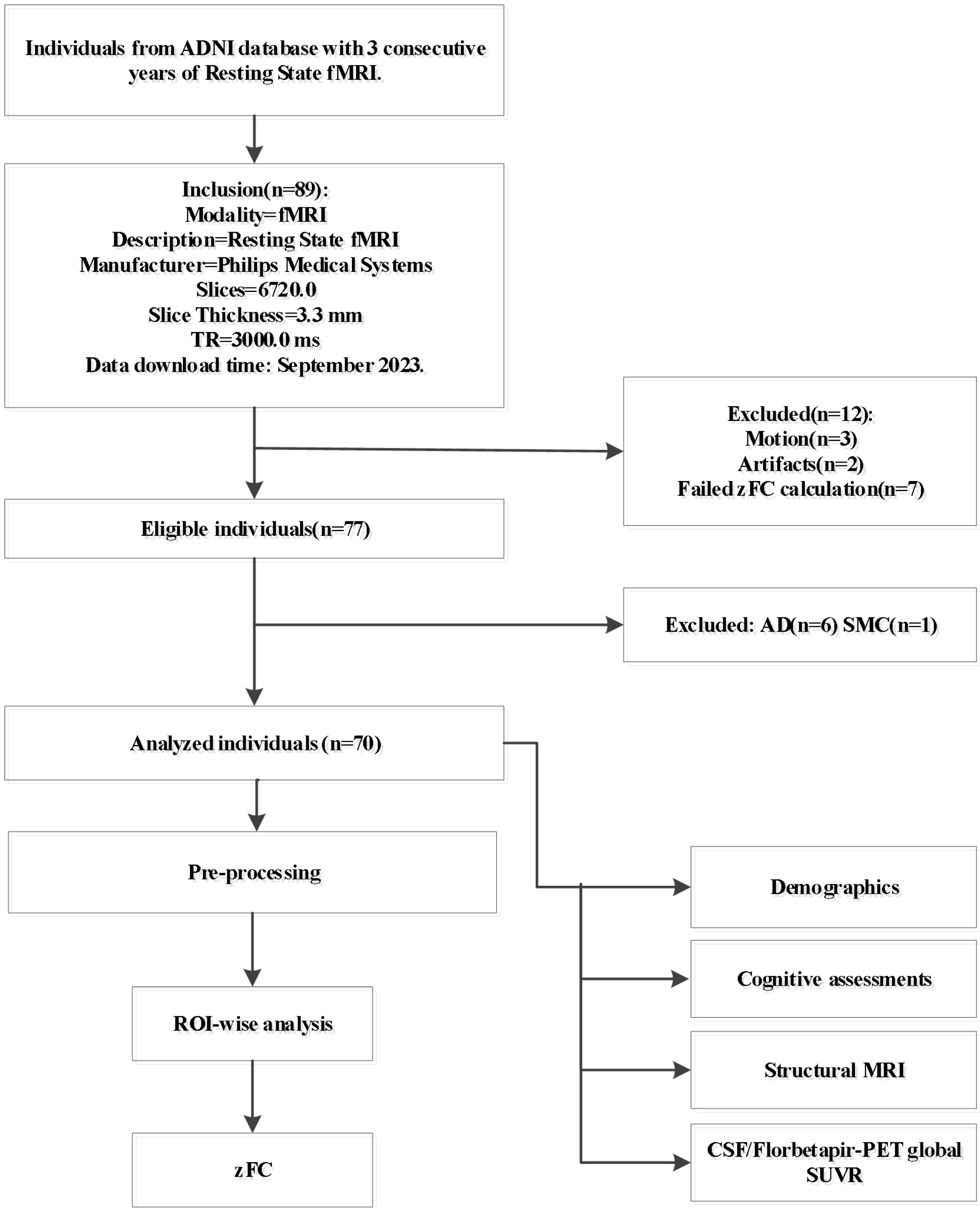

Supplement: Supplementary file 1 [file Data_Sheet_1.PDF]
