# Supplementary material for: Amyloid-β modulates APOE ε4 effects on cognition but not on targeted structural or functional connectivity measures over 2 years in pre-dementia adults
Source: Front Aging Neurosci. 2026 Jul 31;18:1889743. doi: 10.3389/fnagi.2026.1889743 (PMC13472819; doi:10.3389/fnagi.2026.1889743)
Supplement: Supplementary file 2 [file Data_Sheet_2.PDF]

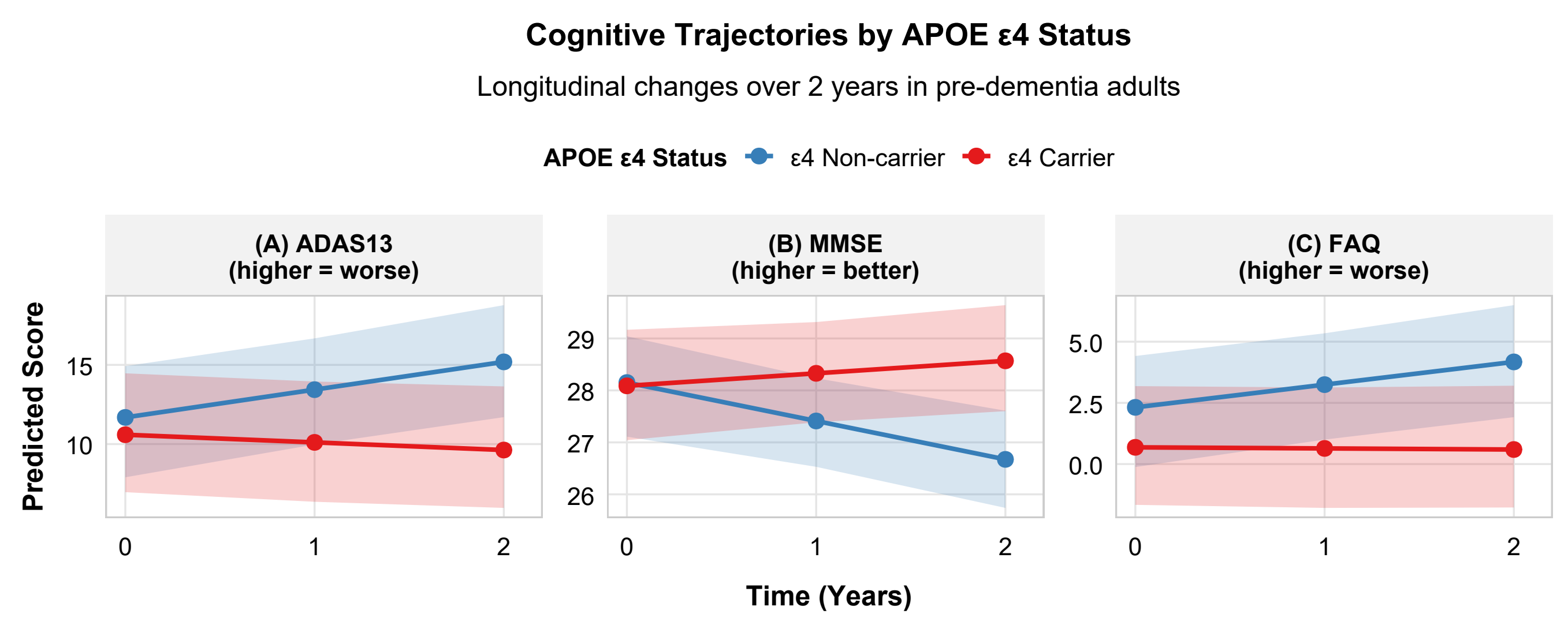
Cognitive Trajectories by APOE ε4 Status
Longitudinal changes over 2 years in pre-dementia adults
APOE ε4 Status
ε4 Non-carrier
ε4 Carrier
(A) ADAS13
(higher = worse)
(B) MMSE
(higher = better)
(C) FAQ
(higher = worse)
Predicted Score
15
10
29
28
27
26
5.0
2.5
0.0
0
1
2
Time (Years)
