# Supplementary material for: Amyloid-β modulates APOE ε4 effects on cognition but not on targeted structural or functional connectivity measures over 2 years in pre-dementia adults
Source: Front Aging Neurosci. 2026 Jul 31;18:1889743. doi: 10.3389/fnagi.2026.1889743 (PMC13472819; doi:10.3389/fnagi.2026.1889743)
Supplement: Supplementary file 3 [file Data_Sheet_3.PDF]

<u>**Supplementary Material**</u>

Contents

Supplementary Figure S1. Flow-chart showing cohort selection, data preprocessing, and analysis process

Supplementary Figure S2. Cognitive trajectories by APOE ε4 status over 2 years in pre-dementia adults (two-way interaction).

Supplementary Table S1. MNI Coordinates of Regions of Interest (ROIs) from the CONN Toolbox

Supplementary Table S2. Longitudinal Associations of APOE ε4 and Amyloid-β Status with Cognitive Decline Over 2 Years

Supplementary Table S3. Longitudinal analysis of structural MRI volumes: three-way interaction and APOE ε4 effects

Supplementary Table S4. Hippocampus-auditory network functional connectivity: longitudinal analysis of the three-way interaction

Supplementary Table S5. Sensitivity analysis: three-way interaction using continuous AV45 SUVR.

Supplementary Table S6. Results of the exploratory mediation analysis testing hippocampal atrophy as a mediator between APOE ε4 and cognitive decline

Supplementary Table S1. MNI Coordinates of Regions of Interest (ROIs) from the CONN Toolbox.

Coordinates are reported in Montreal Neurological Institute (MNI) space. The hippocampus-auditory network functional connectivity analysis included bilateral hippocampus (seed regions) and 12 auditory cortex target regions. ROIs were selected from the CONN default atlas (Harvard-Oxford Subcortical Structural Atlas and the FSL Desikan-Killiany-Tourville Atlas). To minimize multiple comparison errors, we restricted our analysis to core auditory processing areas: Heschl's gyrus (primary auditory cortex), anterior superior temporal gyrus, and posterior superior temporal gyrus (auditory association cortices).

Region	Side	x (mm)	y (mm)	z (mm)
**Hippocampus (Seed)**	Left	-25.18	-23.19	-13.81
**Hippocampus (Seed)**	Right	26.50	-20.96	-14.25
Heschl's Gyrus (TTG)	Left	-45.20	-20.32	7.19
Heschl's Gyrus (TTG)	Right	46.11	-17.40	6.97
Anterior STG	Left	-56.17	-3.91	-7.97
Anterior STG	Right	57.50	-0.76	-10.17
Posterior STG	Left	-62.29	-29.17	3.80
Posterior STG	Right	61.34	-23.99	1.57

AI 生成

STG = Superior Temporal Gyrus; MTG = Middle Temporal Gyrus; ITG = Inferior Temporal Gyrus; TTG = Transverse Temporal Gyrus. Coordinates are rounded to two decimal places.

Supplementary Table S2. Longitudinal Associations of APOE ε4 and Amyloid-β Status with Cognitive Decline Over 2 Years

Outcome	Term	Beta	SE	t	P	FDR
ADAS13	Time*APOEε4	-2.102	0.640	-3.284	0.001	**0.008**
	Time*:A+	0.883	0.663	1.331	0.186	0.325
CDRSB	Time*APOEε4	-0.283	0.150	-1.884	0.062	0.079
	Time*:A+	0.318	0.149	2.127	0.036	0.249
FAQ	Time*APOEε4	-0.979	0.400	-2.449	0.016	**0.037**
	Time*:A+	0.571	0.406	1.408	0.162	0.325
LDELTOTAL	Time*APOEε4	0.109	0.494	0.222	0.825	0.825
	Time*:A+	0.331	0.492	0.673	0.502	0.586
MMSE	Time*APOEε4	0.946	0.302	3.129	0.002	**0.007**
	Time*:A+	-0.375	0.313	-1.198	0.233	0.327
MOCA	Time*APOEε4	0.578	0.314	1.844	0.068	0.079
	Time*:A+	-0.448	0.315	-1.424	0.157	0.325
RAVLT_immediate	Time*APOEε4	2.001	0.918	2.181	0.031	0.054
	Time*:A+	-0.374	0.935	-0.400	0.690	0.690

Note: Estimates (β) represent the difference in annual rate of change (slope) per unit increase in the predictor. Time*APOEε4 represents the interaction between time and APOEε4 carrier status (reference: non-carriers). Time*A+ represents the interaction between time and amyloid-β status (reference: Aβ-negative). All models included random intercepts for participants and were adjusted for baseline age, sex, years of education, FDG-PET SUVR, and plasma p-tau181 levels. Bold text indicates statistical significance at $P < 0.05$ after FDR correction.

SE, standard error; t, t-statistic; P, unadjusted P-value; P_FDR, false discovery rate-corrected P-value; A+, amyloid-β positive. ADAS13, Alzheimer’s Disease Assessment Scale-Cognitive Subscale 13 items; CDRSB, Clinical Dementia Rating Sum of Boxes; FAQ, Functional Activities Questionnaire; LDELTOTAL,Logical Memory II delayed recall;MMSE, Mini-Mental State Examination; MoCA, Montreal Cognitive Assessment; RAVLT, Rey Auditory Verbal Learning Test.

Supplementary Table S3. Longitudinal analysis of structural MRI volumes: three-way interaction and APOE ε4 effects.

Region	Beta_3way	SE_3way	t_value	P_value	P_FDR
Hippocampus	114.492	80.225	1.427	0.157	0.209
Hippocampus_APOEε4	-30.511	62.536	-0.488	0.627	0.730
WholeBrain	8889.012	4258.659	2.087	0.039	0.122
WholeBrain_APOEε4	-6591.370	3301.411	-1.997	0.049	0.122
Ventricles	-2600.646	1014.696	-2.563	0.012	0.071
Ventricles_APOEε4	-348.662	814.674	-0.428	0.670	0.730
Entorhinal	203.300	102.769	1.978	0.051	0.122

AI 生成

Entorhinal_APOEε4	-118.559	79.944	-1.483	0.141	0.209
MidTemp	725.79	263.023	2.759	0.007	0.070
MidTemp_APOEε4	-345.730	204.606	-1.690	0.094	0.189
ICV	6964.401	4540.491	1.534	0.128	0.209
ICV_APOEε4	-825.942	3442.256	-0.240	0.811	0.811

Linear mixed-effects models were used to examine the associations between APOE4, amyloid-β (Aβ) status, and longitudinal changes in regional brain volumes over 2 years. All models were adjusted for age, sex, education, FDG uptake, plasma p-tau, and intracranial volume (ICV; except for ICV itself). The table reports two sets of statistics: (1) the **three-way interaction term (Time × APOEε4 × Aβ status)**, testing whether Aβ pathology moderates the effect of APOE4 on atrophy rates; and (2) the **two-way interaction (Time × APOEε4)**, representing the overall effect of APOE4 on atrophy regardless of Aβ status.

Following false discovery rate (FDR) correction for multiple comparisons across six structural regions, **no significant three-way interactions survived** (all P_FDR> 0.05). Nominal trends (uncorrected $P < 0.05$, denoted by †) were observed for ventricular expansion and middle temporal gyrus atrophy, suggesting a potential weak modulation by Aβ pathology in these regions. Similarly, the overall effect of APOE4 (Time × APOE4) on whole brain volume showed only a nominal trend ($P = 0.049$) that did not survive multiple testing correction.

The absence of robust structural interactions over this relatively short 2-year interval, despite significant cognitive associations, aligns with the temporal trajectory of Alzheimer's disease pathophysiology, wherein measurable cognitive decline often precedes detectable macrostructural gray matter atrophy. Positive three-way interaction coefficients indicate relative volume preservation among Aβ-positive ε4 carriers, consistent with the cognitive protective pattern. Negative coefficients for ventricular expansion indicate less enlargement among Aβ-positive ε4 carriers, also consistent.

Supplementary Table S4. Hippocampus-auditory network functional connectivity: longitudinal analysis of the three-way interaction (Time × APOEε4 × Aβ status)

FC	Beta_3way	SE_3way	t_value	P_value	P_FDR
R Hippo-L Post.STG	-0.238	0.094	-2.536	0.013	0.151
R Hippo-R Heschl's	0.182	0.097	1.87	0.064	0.324
L Hippo-R Post. STG	-0.183	0.107	-1.72	0.088	0.324
L Hippo-R Ant.STG	-0.140	0.091	-1.538	0.127	0.324
R Hippo-R Post. STG	-0.167	0.111	-1.506	0.135	0.324
R Hippo-L Ant.STG	-0.109	0.117	-0.933	0.353	0.706
L Hippo-L Post. STG	-0.074	0.110	-0.674	0.502	0.776
R Hippo-L Heschl's	-0.055	0.095	-0.578	0.564	0.776
L Hippo-R Heschl's	0.035	0.106	0.329	0.743	0.776
L Hippo-L Ant.STG	-0.038	0.114	-0.329	0.743	0.776
L Hippo-L Heschl's	-0.034	0.103	-0.326	0.745	0.776

AI 生成

R Hippo-R Ant.STG	0.031	0.108	0.285	0.776	0.776

Resting-state functional connectivity (FC) was analyzed for 12 predefined hippocampus-auditory cortex ROI pairs using linear mixed-effects models adjusted for age, sex, education, FDG, p-tau, and intracranial volume. The table reports the coefficient estimates (β) for the three-way interaction term, reflecting how amyloid-β (Aβ) pathology moderates the longitudinal effect of APOEε4 on FC strength.

After false discovery rate (FDR) correction for 12 ROI comparisons, no connections survived as statistically significant (all P_FDR> 0.05). A nominally significant association (uncorrected P < 0.05) was observed for R Hippo-L Post. STG connection, suggesting a potential localized alteration where Aβ positivity may modulate APOEε4-related FC changes over time. The overall lack of robust FC interactions over the 2-year follow-up, despite significant cognitive findings, suggests that in this prodromal cohort, cognitive decline manifests prior to widespread disruptions in hippocampus-auditory network synchrony, or that FC metrics may require longer observation windows to capture early network-level adaptations. The negative three-way interaction coefficient for the right hippocampus–left posterior STG connection suggests greater connectivity decline among Aβ-positive ε4 carriers, which is opposite to the cognitive protective pattern.

Supplementary Table S5. Sensitivity analysis: longitudinal three-way interaction (Time × APOE ε4 × continuous AV45 SUVR) on cognitive decline.

Outcome	Beta	SE	t	P	P_FDR
ADAS13	-10.4625	3.0187	-3.4659	0.0005	**0.0018**
MMSE	4.7307	1.4211	3.3288	0.0009	**0.0020**
CDRSB	-2.3446	0.7268	-3.2259	0.0013	**0.0022**
FAQ	-2.0669	2.0443	-1.0111	0.3120	0.3120
MOCA	5.2587	1.4993	3.5074	0.0005	**0.0018**
LDELTOTAL	4.3916	2.5056	1.7527	0.0797	0.1115
RAVLT_immediate	6.7935	4.6121	1.4730	0.1408	0.1642

Note: Linear mixed-effects models were used to examine the three-way interaction of Time × APOE ε4 × continuous AV45 SUVR (centered) on longitudinal cognitive trajectories. AV45 SUVR was mean-centered before analysis. All models were adjusted for age, sex, education, FDG-PET SUVR, and plasma p-tau181 levels, with random intercepts for participants. P_FDR values were calculated using the Benjamini-Hochberg procedure across all seven cognitive outcomes. Bold values indicate P_FDR < 0.05. All seven measures showed directionally concordant interactions with the dichotomized Aβ status analysis (see Supplementary Table S2).

Supplementary Table S6. Results of the exploratory mediation analysis testing hippocampal atrophy as a mediator between APOE ε4 and cognitive decline (ADAS13).

AI 生成

Path	β	SE	t value	P value	95%CI
c: Total effect (APOEε4 → ADAS13)	-2.236	0.668	-3.34	0.001	[-3.546, -0.925]
a: APOEε4 → Hippocampus	13.686	34.528	0.40	0.692	[-53.989, 81.360]
b: Hippocampus → ADAS13 (controlling APOEε4)	-0.001	0.000	-3.21	0.002	[-0.002, -0.000]
c': Direct effect (APOEε4 → ADAS13)	-1.785	0.642	-2.78	0.006	[-3.044, -0.526]
a×b: Indirect effect (APOEε4 → Hippocampus → ADAS13)	-0.014	—	—	>0.05*	[−0. 141, 0. 086]†
Proportion Mediated		—	—	—	—

Note: β, unstandardized coefficient; SE, standard error; CI, confidence interval. All models were adjusted for baseline age, sex, education, FDG, plasma p-tau, and intracranial volume. Paths a, b, c, and c' are based on linear mixed-effects models.

† Bootstrap 95% confidence interval, based on 2,000 resamples. A confidence interval that includes zero indicates a non-significant indirect effect (P > 0.05).

AI 生成
